# High Glycemic Load Is Associated with Cognitive Decline in Apolipoprotein E ε4 Allele Carriers

**DOI:** 10.3390/nu12123619

**Published:** 2020-11-25

**Authors:** Mélissa Gentreau, Michel Raymond, Virginie Chuy, Cécilia Samieri, Catherine Féart, Claire Berticat, Sylvaine Artero

**Affiliations:** 1 Neuropsychiatry: Epidemiological & Clinical Research, University of Montpellier, INSERM, 34093 Montpellier, France; melissa.gentreau@inserm.fr; 2 ISEM, University of Montpellier, CNRS, EPHE, IRD, 34095 Montpellier, France; michel.raymond@umontpellier.fr (M.R.); claire.berticat@umontpellier.fr (C.B.); 3 Bordeaux Population Health Research Center, University of Bordeaux, Inserm, UMR 1219, F-33000 Bordeaux, France; virginie.chuy@u-bordeaux.fr (V.C.); cecilia.samieri@u-bordeaux.fr (C.S.); catherine.feart-couret@u-bordeaux.fr (C.F.)

**Keywords:** cognition, glycemic load, carbohydrate, sugar, diet, apolipoprotein E4, dementia

## Abstract

Recent evidence suggests that a high glycemic load (GL) diet is a risk factor for dementia, especially among apolipoprotein E ε4 allele (*APOE4*) carriers, while its association with cognitive decline is poorly known. Here, we investigated the association of high-GL meals with cognitive decline in older adults during a 12-year follow-up, according to their *APOE4* carrier status. We used random-effect models and data from 2539 elderly participants from the Three-City study who completed a food frequency questionnaire (FFQ) to longitudinally assess the association of GL with changes in different cognitive domains (verbal fluency, visual memory, attention, visual motor processing speed, episodic memory). In *APOE4* carriers, afternoon snack with high GL was significantly associated with cognitive decline in visual memory, episodic memory, and global cognition compared with *APOE4* non-carriers. This study suggests a detrimental association between a high-GL diet and cognitive decline. The promotion of a low GL diet as a target to prevent cognitive decline in high-risk populations deserves more research.

## 1. Introduction

In aging societies, cognitive health has become a major concern. Currently, there is no effective pharmacological treatment for dementia and cognitive decline. Therefore, the identification of modifiable risk factors that could delay or prevent cognitive decline has become a key public health research axis [1]. Promisingly, some evidences suggest that cognitive function integrity may be promoted by healthy diets and some nutritional factors [2]. Moreover, diet can interact with the apolipoprotein E ε4 allele (*APOE4*) [3,4], the main genetic risk factor of dementia.

*APOE4* carriers represent 14 to 15% of the general population and are quite stable [5,6]. They had three fold-increased risk of Alzheimer’s disease than *APOE4* non-carriers [7] and represent around 65% among Alzheimer’s disease patients [6,8]. ApoE is mostly involved in lipid transport, cholesterol homeostasis and synaptic plasticity but also mitochondrial function and insulin signaling [9]. Specifically, *APOE4* status may modulate the metabolic response to diet interventions [10]. Notably, *APOE4* carriers show early deficits in cerebral glucose metabolism [11,12], and recent findings strongly suggest that *APOE4* carriers are less sensitive to insulin [8,13]. A causal link between insulin metabolism and the pathogenesis of dementia has been suggested [14,15]. Thus, optimizing blood glucose and insulin responses through nutritional interventions based on a low glycemic load (GL) diet could be particularly relevant to prevent cognitive decline in these individuals.

In this context, our recent work highlighted that high-GL diet, which reflects an elevated intake of refined carbohydrates, increases the risk of dementia in *APOE4* carriers [16]. Yet, the impact of the diet GL on long-term cognitive performance, especially in the different cognitive domains (verbal fluency, visual memory, attention, visual motor processing speed, episodic memory), is poorly understood. Only one longitudinal study found that the diet GL was significantly related to lower decline in verbal ability [17], and no study tested the potential interaction with *APOE4* status. Cognitive decline precedes the onset of dementia but may also be a marker of brain aging. The use of cognitive tests enables the identification of the different cognitive functions effectively associated with the GL diet and which ones are the most affected.

Moreover, meals have a different nutritional composition that will not bring the same glycemic response [18]. During meals, carbohydrates are rarely ingested alone, and their degradation and absorption rates during digestion are modified by the other macronutrients (i.e., matrix effect). The glycemic response will be more important after intake of a meal rich in refined carbohydrates, and poor in fibers, fat, and proteins [19,20]. Thus, meals that are generally higher in refined carbohydrates, such as breakfast and snacks, may induce a higher glycemic response and consequently increase the risk of cognitive decline. However, the effect of the GL of the different meals on the different cognitive domains has been rarely studied.

In the present study, we determined whether high-GL diet was associated with cognitive changes in the different cognitive domains during a 12-year follow-up using data from a large population-based prospective cohort study (the Three-City study). We evaluated the role of *APOE4* status on the association between GL and cognitive changes, and the impact of the GL of each meal type (daily, breakfast, lunch, afternoon snack, and dinner) on this association.

As we previously observed an interaction between GL and *APOE4* only for the afternoon snack (snack between lunch and dinner) [16], we hypothesized that, in *APOE4* carriers, the afternoon -snack GL might be associated with a greater decline in cognitive performance over time.

## 2. Materials and Methods

### 2.1. Study Sample

**Figure 1:**
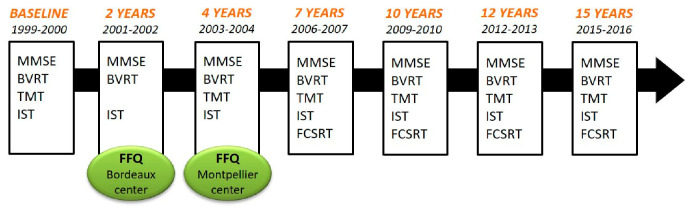
Time frame of the three-city study. Neuropsychological evaluations and dementia diagnoses were made at each follow-up visit. Participants completed the FFQ at the 2-year follow-up visit (Bordeaux center) or at the 4-year follow-up visit (Montpellier center). BVRT, Benton Visual Retention Test; FCSRT, Free and Cued Selective Reminding Test; FFQ, Food Frequency Questionnaire; IST, Isaacs Set Test; MMSE, Mini Mental State Examination; TMT, Trail Making Test.

**Figure 2:**
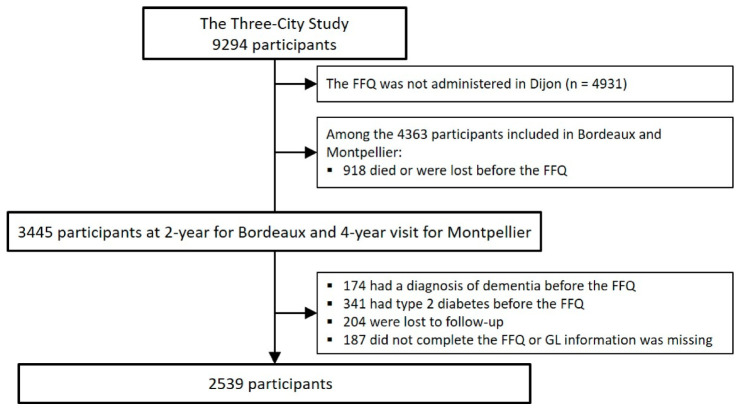
Three-City study flow chart.

Participants (n = 9294) were recruited from the Three-City (3C) cohort of community-dwelling individuals, aged 65 years and over, from the electoral rolls of three French cities (Bordeaux, n = 2104; Dijon, n = 4931; and Montpellier, n = 2259), enrolled between 1999 and 2001. A detailed description of the 3C study has been provided elsewhere [21]. The Ethical Committee of the University Hospital of Kremlin-Bicêtre (France) approved the 3C protocol (Project no. 99-28, June 1999), and all participants signed an informed consent. Participants from the Bordeaux and Montpellier 3C centers had face-to-face interviews with trained nurses and neuropsychologists at baseline, and at 2, 4, 7, 10, 12 and 15 years of follow-up. Standardized questionnaires were used to collect data on socio-demographic, clinical and lifestyle characteristics. A Food Frequency Questionnaire (FFQ) was administered to participants from the Bordeaux and Montpellier 3C centers at the 2-year follow-up and 4-year follow-up visit, respectively (Figure 1). Thus, the baseline of the present analyses was the 2-year follow-up visit for Bordeaux and the 4-year follow-up visit for Montpellier. This subsample has been previously described [16]. Participants with prevalent dementia at the baseline of the present analyses were excluded (n = 174). Participants with type 2 diabetes (defined as treated diabetes, fasting glycemia > 7mmol.L^−1^, and self-reported) at baseline were excluded from the analyses (n = 341, 18% were *APOE4* carriers) because they were at risk of cognitive decline and followed a low GL diet (due to their treatment and diet changes). Finally, the maximum sample for the main analyses included 2539 participants (Figure 2).

### 2.2. Dietary Data

Dietary data were collected using the 148-item FFQ. The FFQ was divided into breakfast, lunch, dinner, and snacks between meals, and was described elsewhere [16]. For each FFQ item (i.e., food/beverage categories), the GL was calculated as the product of the glycemic index (GI) and the amount of available carbohydrates in a standard serving size, divided by 100 [22]. Foods with low carbohydrate content (e.g., meat, fish, fats, vegetables) were assigned a null GL value. GI values and serving sizes were obtained from the International Table of Glycemic Index [23] and internet updates [24], using glucose as a reference. Then, the GL of each item was multiplied by the reported frequency of that item per week. Finally, the sum of this last value for all the items consumed during a meal gave the total GL for breakfast, lunch, afternoon snack (“goûter” in French, corresponding to a snack between lunch and dinner), and dinner. The sum of all GL gave the daily GL. Energy intakes (EI) were obtained from the Anses-Ciqual database [25]. The EI per serving of each item was multiplied by the reported frequency. Then, the sum of the EI for the items consumed during a meal gave the EI for breakfast, lunch, afternoon snack, and dinner. The sum of all EI gave the daily EI.

### 2.3. Neuropsychological Evaluation and Dementia Diagnosis

A battery of cognitive tests was administered by trained neuropsychologists with standard procedures at baseline and at each follow-up (2, 4, 7, 10, 12 and 15 years). The evaluation took place at any time of the day, depending on the time of participants’ enrollment. Cognitive tests evaluated different cognitive domains (Figure 1). The Isaacs Set Test (IST) [26] measures verbal fluency: participants had 30 s to generate as many words as possible within a given semantic category (animals, colors, fruits and cities). The Benton Visual Retention Test (BVRT) [27] evaluates immediate visual memory. The scores range from 0 to 15. The Trail Making Test, forms A and B (TMTA and TMTB) [28], evaluates attention and visual motor processing speed. TMTB also assesses executive functions. The score is the task execution time (time in seconds). Thus, a higher score corresponds to a lower cognitive performance. The French version [29] of the Free and Cued Selective Reminding Test (FCSRT) [30] evaluates verbal episodic memory [31]. Free and cued recalls are repeated three times, and the delayed recall phase is performed 20 min later. Given the non-normal distribution of the “total immediate recall score” and the “total delayed recall score”, only the “free immediate recall score” (FIRS, sum of the number of words retrieved at the three free recall trials) and the “free delayed recall score” (FDRS, number of words retrieved at the delayed free recall trial) were used. Finally, the widely used Mini Mental State Examination (MMSE) [32] was used as an index of global cognitive performance. The score ranges from 0 to 30.

All cognitive tests were administered at baseline and at each follow-up, except the TMT that was not proposed at the 2-year follow-up visit, and the FCSRT that was administered only at the 7-, 10-, 12- and 15-year follow-up visit. For each cognitive test, participants with at least two measures during the follow-up where included in the analyses. Finally, because of sparse missing data, the analyses involved, in *APOE4* non-carriers and *APOE4* carriers respectively: 1815 and 401 participants for IST, 1794 and 401 for BVRT, 1612 and 348 for TMTA, 1593 and 348 for TMTB, 1351 and 278 for FIRS, 1348 and 275 for FDRS, 1831 and 409 for MMSE.

To identify prevalent and incident dementia cases, according to their neuropsychological test scores, participants with suspected dementia, at baseline and at each follow-up, were examined by a neurologist. Then, an independent committee of neurologists evaluated all potential cases of dementia to obtain a consensus on the diagnosis and etiology based on the Diagnostic and Statistical Manual of Mental Disorders, Fourth Edition [33].

### 2.4. Covariates

Apolipoprotein E genotyping was described elsewhere [34]. *APOE4* carriers had at least one ε4 allele. Education level was defined as no school, primary school, high school, or graduated level. Body mass index (BMI) was calculated as weight (kg)/height^2^ (m). Hypertension was defined by systolic blood pressure ≥ 140 mmHg, or diastolic blood pressure ≥ 90 mmHg, or use of antihypertensive drugs [35]. History of stroke was established using standardized questions. Cardiovascular history included history of myocardial infarction, coronary surgery, coronary angioplasty, and arterial surgery of the legs for arteritis. Hypercholesterolemia was defined as total cholesterol ≥ 6.2 mmol.L^−1^. Depressive symptomatology was evaluated with the Center for Epidemiological Studies-Depression (CES-D) scale [36] using the recommended French cut-off scores of 17 and 23 for clinically relevant depressive symptom burden in older men and women, respectively [37]. Smoking status (never, past, or current) was also investigated. The Mediterranean-like diet score was used to control for diet quality, as previously described [16]. Physical activity was defined as none, or low/regular [38].

### 2.5. Statistical Analyses

#### 2.5.1. Main Analyses

Linear random-effect models were used to evaluate the association between GL and cognitive score changes over the 12-year follow-up. We used the *lme* function from the *nlme* R package with random intercept and slope (time), and unstructured covariance structure. Normal distributions of cognitive scores were checked with histograms. To normalize distributions, TMT scores were transformed using the natural logarithm function. The linearity assumption between cognitive score and GL was assessed by testing the loglikehood difference between two models: one model with GL tertiles as continuous variable and one model with GL tertiles as categorical variable. As the relationship between GL and cognitive scores was nonlinear, cut-offs were chosen a priori according to tertiles. The GL categories were defined as low, middle and high tertile. For daily GL, the cut-offs were: <93, 93 to <119 and ≥119 per day. For breakfast GL, the cut-offs were: <19, 19 to 28, ≥28 per day. For lunch GL, the cut-offs were: <29, 29 to 36, ≥36 per day. For afternoon snack, the cut-offs were: null value, 0 to 10, ≥10 per day. For dinner, the cut-offs were: <28, 28 to 36, ≥36 per day.

The β coefficient for the GL tertiles represents the association of GL with each baseline mean cognitive score. The β coefficient for the GL tertile × time interaction represents the association of GL with the cognitive score changes over time, expressed as a score change per year. For MMSE, BVRT, IST and FCSRT, a negative β coefficient indicates a decrease in the cognitive change slope. For TMT, a negative β coefficient indicates an increase in the cognitive change slope.

These models were adjusted for potential confounding factors (based on literature data) including time, center, age, sex, education level, *APOE4*, EI, BMI, hypertension, cardiovascular history, stroke history, hypercholesterolemia, CES-D score, smoking status, Mediterranean-like diet, and physical activity. For each confounding factor, the interaction with time was tested and was added it to the models when significant (i.e., center × time, age × time, sex × time, and education level × time).

To test the *APOE4* effect on the relationship between GL and cognitive performance, an interaction term with *APOE4* status was added to the GL tertile and GL tertile x time in each model. In that case, the β coefficient for the GL tertile × time × *APOE4* interaction represents the additive effect of *APOE4* status to the main effect (i.e., the β coefficient for the GL tertile × time alone) on the cognitive score changes over time, expressed as a score change per year.

As some models showed a significant GL tertile × time × *APOE4* interaction, analyses were stratified by *APOE4* status (i.e., *APOE4* non-carriers and *APOE4* carriers).

#### 2.5.2. Sensitivity Analyses

In sensitivity analyses, the same analyses were repeated after excluding individuals with incident dementia (n = 337).

#### 2.5.3. Imputation of Missing Values

Data on physical activity were missing for 14%, on Mediterranean-like diet for 7%, and on hypercholesterolemia for 3% of the main study sample. Incomplete variables were imputed by multiple imputation using the *mice* R package [39], as previously described [16].

All statistical analyses were carried out with the R 3.4.3 software [40].

## 3. Results

### 3.1. Population Characteristics

Table 1 describes the demographic and clinical characteristics of the 2539 participants selected for this study and grouped according to their daily GL tertile (low, middle and high). The participants’ mean age at the time of FFQ completion (i.e., baseline) was 76 years ± 4.9. The high daily GL group was characterized by a lower percentage of women, older age, higher level of physical activity, higher Mediterranean-like diet score, higher EI, lower BMI, and lower percentage of participants with hypertension. Participants from the Montpellier center were more numerous in the high daily GL tertile.

Participants consuming afternoon snack were more likely to be women. They had a higher energy intake but they also tended to practice more often physical activity, had a better Mediterranean diet score and had a slightly lower BMI (Table S1).

### 3.2. Main Analyses

The GL tertile × time × *APOE4* interaction was not significant for daily GL, lunch GL, and dinner GL (Table S2). The interaction high breakfast GL x time x *APOE4* was associated with reduced BVRT, the interaction middle breakfast GL × time × *APOE4* was associated with reduced MMSE. As expected, the interaction afternoon snack GL × time × APOE4 was significant. The interaction high afternoon snack GL × time × APOE4 was associated with decreased of cognitive score through time regarding IST, TMTA, FIRS, and MMSE.

#### 3.2.1. Association between GL and Cognitive Changes in APOE4 Non-Carriers (12-Year Follow-up)

In *APOE4* non-carriers (Table 2), the middle daily GL tertile was significantly associated with better TMTA performance over time (−0.30 s per year). Likewise, the middle and high-GL tertiles for lunch were associated with improved TMT performance over time. The GL high tertile for lunch was associated with lower baseline scores for IST, BVRT, and TMTA. Breakfast, afternoon snack and dinner GL did not seem to be related to cognitive changes. However, the high-GL tertile for dinner was associated with a lower baseline TMT scores compared with the low tertile (i.e., the reference) (−3.43 s for TMTA and −7.76 s for TMTB).

#### 3.2.2. Association between Glycemic Load and Cognitive Changes in APOE4 Carriers (12-Year Follow-up)

In *APOE4* carriers (Table 3), the middle GL tertile for breakfast was associated with decreasing MMSE scores (−0.14 points per year). The afternoon snack GL was associated with cognitive decline, particularly between middle tertile and BVRT score (−0.07 points per year), high tertile and FDRS score (−0.18 points per year), and high tertile and MMSE score (−0.16 points per year).

### 3.3. Sensitivity Analyses

Participants who developed dementia during the 12-year follow-up (n = 337) were older (78.1 vs. 75.6 years; *p* < 0.0001, Table S3) at the time of FFQ completion than those who remained free from dementia. Participants with incident dementia were more likely to be women and to be *APOE4* carriers. At baseline, they had lower MMSE and Mediterranean-like diet scores. In sensitivity analyses that evaluated the association between GL tertiles and cognitive performance by excluding participants with incident dementia (Tables S4 and S5), the afternoon snack GL × time × *APOE4* interaction was no longer significant.

In *APOE4* non-carriers, the results of the sensitivity analyses were qualitatively comparable to those of the main analyses, except for the association between lunch GL tertile and baseline IST score that was no longer significant. In *APOE4* carriers, no association remained significant.

Taken together, these results suggest that in *APOE4* carriers, the afternoon snack GL is associated with cognitive decline, but not in the sample without participants with incident dementia.

## 4. Discussion

Our results suggest that high afternoon snack GL is associated with cognitive decline in visual memory (BVRT), episodic memory (FCSRT) and global cognition (MMSE) in *APOE4* carriers during a 12-year follow-up.

To our knowledge, no study has reported the effect of the GL of different meals on the long-term cognitive performance. Cross-sectional studies showed that high-GL diet is related to poorer cognitive performance. Specifically, three epidemiological studies found that high-GL diet is associated with lower global cognition [41,42,43], and two with poorer episodic memory [42,44]. One longitudinal study showed that a high-GL diet is associated with reduced verbal ability [17]. The 1946 British birth cohort study also found that higher GI diet at the age of 53 years was associated with lower episodic memory and processing speed at the age of 69 years, although these associations did not remain significant after adjustment for education and cognitive abilities at the age of 15 years [45]. In the Chinese Longitudinal Health Longevity Survey, consumption of sugar “almost every day” was associated with a 17% increase in the risk of cognitive impairment during the 15-year follow-up [46].

Regarding the effect of *APOE4* status on the diet GL with cognitive performance interaction, Gardener and collaborators reported that high-GL diet is associated with lower episodic memory in *APOE4* non-carriers and with lower attention capacity in *APOE4* carriers [47]. Hanson and collaborators also found a differential effect of a meal with high GI on cognition according to *APOE4* carrier and cognitive status (normal cognition vs. cognitive impairment) [48]. Specifically, when switching from the high-GI meal to the low-GI meal, the delayed memory composite score was better in *APOE4* carriers with normal cognition than in *APOE4* non-carriers. In addition, *APOE4* carriers with cognitive impairment had a better executive function composite score than *APOE4* non-carriers with normal cognition, when switching from the high-GI meal to the low-GI meal.

Here, we found that the interaction between afternoon snack GL and *APOE4* was associated with cognitive decline, suggesting an underlying dementia-specific mechanism between *APOE4* and carbohydrate metabolism. Our findings are consistent with our previous work showing that the afternoon snack GL and *APOE4* interaction is associated with dementia risk [16]. They also highlight that high afternoon snack GL might contribute to dementia development by affecting specific cognitive domains (visual memory and episodic memory) in *APOE4* carriers.

The relationship between afternoon snack GL and cognitive decline among *APOE4* carriers could be explained by a biological mechanism. First, the afternoon snack is the meal with the highest GL foods and the breakfast is the second one [16]. The more a meal has a high GL the more will be the spike of glycemia and insulinemia. Thus, a chronic exposure to high-GL diet, like snacking, could promote the development of insulin resistance and impaired glucoregulation [49,50], trough oxidation stress and inflammation [51,52]. In addition, it has been demonstrated that *APOE4* carriers have poor brain glucose metabolism [11,12], and tend to be insulin resistant [8,9]. Indeed, in animal models, ApoE4 impairs insulin signaling [13]. This hypothesis is supported by several epidemiological studies [14,49,53,54]. For examples, in older adults with poor glucoregulation, GL was inversely associated with global cognitive function and figural memory [53]. In the Framingham Heart Study, elevated glycemia in midlife was associated with more severe Alzheimer’s disease pathology in *APOE4* carriers [54].

Conversely, we found that the daily GL middle tertile was associated with better attention and visual motor processing speed (TMTA) over time, and that the middle and high lunch GL tertiles were associated with better executive function (TMT) over time. One interventional study showed that dietary carbohydrates could increase cognitive performance in healthy elderly subjects with poor memory or poor β cell function, independently of glucoregulation [55]. Lunch is the main meal of the day and the lunch GL was strongly correlated with the overall daily GL (r = 0.64; *p* < 0.0001). In France, the most frequent components of lunch are vegetable and legumes (i.e., fibers) and, meat that have a null GL value, dairy products that have a relatively low GL value, and bread and cereals [10]. Although bread and cereals have a quite high GL, they are rarely ingested alone in the context of a meal. The ingestion of cereals with fat and fiber decreases the glycemic response [19,20]. Thus, a healthy meal composed of vegetables, fruits and some cereals could be beneficial for the cognitive performance. Indeed, it has been largely demonstrated that the Mediterranean diet is a protective factor against cognitive decline [2]. Moreover, lunch, dinner, and the daily GL are highly correlated with EI. Thus, the beneficial effects of high GL on cognitive performance could be explained by the maintenance of an adequate EI needed for good health in the elderly.

Our current study presents some limitations. First, we cannot exclude the possibility that the absence of significant associations between the *APOE4* × afternoon snack GL interaction and cognitive decline in the sensitivity analysis without participants with incident dementia is due to lack of statistical power. Indeed, by excluding participants who developed dementia (13%), 18% of *APOE4* carriers were removed from the sample (Table S3). Second, our GL estimations relied on a FFQ that is less detailed than other dietary assessments. However, GL estimations from the FFQ were previously validated by 24-h dietary recalls [16]. Third, as the dietary assessment was carried out only at baseline, we cannot exclude that participants may have changed their diet during the follow-up. The major strength of our study is the large sample size and the longitudinal design with repeated cognitive assessments over time that allowed monitoring the cognitive decline kinetics. In addition, the cognitive assessments covered large cognitive domains: verbal fluency, visual memory, attention, visual motor processing speed, episodic memory, and global cognition.

In this prospective study, high afternoon snack GL was associated with cognitive decline in *APOE4* carriers. These results must be now replicated. Nevertheless, they might help to develop intervention strategies with the aim of delaying or preventing cognitive decline. Our study also invites to better consider in future studies the role of the interaction between *APOE4* and refined carbohydrate-rich diet on cognition.

## Figures and Tables

**Table 1:** Description of the study population (n = 2539).

Characteristics Mean (SD) or n (%)	Daily Glycemic Load Tertiles			
	Low (<93) n = 846	Middle (93 to <119) n = 845	High (≥119) n = 848	*p* Value ^1^
Montpellier center	342 (40.4)	390 (46.2)	527 (62.1)	<0.0001
Age (years)	75.9 (4.8)	75.5 (4.8)	76.4 (4.9)	0.038
Women	572 (67.6)	512 (60.6)	515 (60.7)	0.003
Education level				0.19
No school	231 (27.3)	216 (25.6)	208 (24.5)	
Primary school	237 (28)	265 (31.4)	224 (26.4)	
High school	192 (22.7)	183 (21.7)	210 (24.8)	
Graduated	185 (21.9)	178 (21.1)	204 (24.1)	
MMSE (IQR)	28 (27-29)	28 (27-29)	28 (27–29)	0.805
*APOE4* carriers	142 (16.8)	156 (18.5)	144 (17)	0.539
BMI (kg/m^2^)	26.1 (4.1)	25.6 (3.9)	24.8 (3.4)	<0.0001
Hypertension	521 (61.6)	487 (57.6)	465 (54.8)	0.017
Cardiovascular history	90 (10.6)	82 (9.7)	77 (9.1)	0.555
Stroke history	37 (4.4)	25 (3)	39 (4.6)	0.173
Hypercholesterolemia	464 (54.8)	475 (56.2)	452 (53.3)	0.486
Depressive symptomatology ^2^	74 (8.7)	56 (6.6)	70 (8.3)	0.238
Tobacco use (current or past)	337 (39.8)	310 (36.7)	300 (35.4)	0.15
Mediterranean-like diet	4.3 (1.6)	5 (1.6)	5.5 (1.5)	<0.0001
Physical activity	251 (29.7)	274 (32.4)	317 (37.4)	0.002
Energy intake (kJ/day)	3553.2 (698.8)	4694.5 (656.2)	6379.4 (1476.5)	<0.0001

^1^ Chi-square test for categorical variables, ANOVA or Kruskal–Wallis test for continuous variables. ^2^ evaluated with the Center for Epidemiological Studies-Depression scale. *APOE4*, Apolipoprotein E ε4 allele; BMI, Body Mass Index; IQR Interquartile range; MMSE, Mini Mental State Examination; SD, standard deviation.

**Table 2:** Associations of glycemic load with cognitive changes in *APOE4* non-carriers (12-year follow-up).

Glycemic Load	IST n = 1815	BVRT n = 1794	log(TMTA) n = 1612	log(TMTB) n = 1593	FIRS n = 1351	FDRS n = 1348	MMSE n = 1831
	β (SE)	β (SE)	β (SE)	β (SE)	β (SE)	β (SE)	β (SE)
**Daily**							
Middle	−0.159 (0.654)	−0.1 (0.12)	0.003 (0.026)	0.01 (0.029)	0.649 (0.654)	0.304 (0.285)	−0.096 (0.119)
High	−0.103 (0.82)	−0.129 (0.146)	0.023 (0.032)	−0.004 (0.035)	0.944 (0.757)	0.411 (0.326)	0.03 (0.147)
Middle × time	**0.102** (0.056)	0.008 (0.012)	−0.006 * (0.003)	−0.003 (0.003)	0.015 (0.075)	0.003 (0.034)	0.022 (0.024)
High × time	0.086 (0.055)	0.01 (0.012)	−0.004 (0.003)	−0.001 (0.003)	0.045 (0.073)	0.007 (0.033)	−0.015 (0.024)
**Breakfast**							
Middle	0.608 (0.579)	0.073 (0.109)	-0.033 (0.024)	−0.031 (0.026)	−0.965 (0.604)	0.136 (0.267)	0.058 (0.105)
High	0.136 (0.67)	0.033 (0.122)	0.015 (0.027)	−0.036 (0.029)	−0.31 (0.644)	0.026 (0.28)	0.07 (0.121)
Middle × time	0.031 (0.057)	−0.005 (0.012)	0 (0.003)	0.002 (0.003)	0.136 (0.076)	−0.014 (0.035)	0.023 (0.024)
High × time	0.08 (0.054)	0.015 (0.012)	−0.003 (0.003)	0 (0.003)	0.09 (0.071)	0.01 (0.032)	0.023 (0.023)
**Lunch**							
Middle	−1.312 * (0.6)	−0.07 (0.114)	0.027 (0.025)	0.067 (0.027)	0.292 (0.627)	−0.279 (0.274)	0.017 (0.11)
High	−1.904 ** (0.732)	−**0.324** * (0.133)	0.074 * (0.029)	0.066 (0.032)	0.124 (0.687)	−0.1 (0.297)	−0.054 (0.132)
Middle × time	0.021 (0.057)	−0.008 (0.012)	−0.001 (0.003)	−0.008 ** (0.003)	−0.055 (0.077)	0.026 (0.035)	−0.021 (0.024)
High × time	**0.102** (0.054)	0.011 (0.012)	−0.006 * (0.003)	−0.006 * (0.003)	0.064 (0.072)	0.025 (0.033)	−0.012 (0.023)
**Afternoon snack**							
Middle	−2.125 (5.936)	−0.552 (0.964)	−0.003 (0.216)	−0.07 (0.225)	−2.922 (4.169)	−0.029 (1.732)	−0.328 (1.035)
High	−2.924 (5.978)	−0.61 (0.972)	0.019 (0.217)	−0.047 (0.227)	−3.165 (4.205)	0.16 (1.747)	−0.44 (1.043)
Middle × time	−0.054 (0.054)	−0.006 (0.012)	−0.002 (0.003)	−0.002 (0.003)	0.026 (0.072)	−0.016 (0.033)	−0.016 (0.023)
High × time	0.038 (0.056)	−0.006 (0.012)	−0.002 (0.003)	0.001 (0.003)	0.106 (0.074)	0.001 (0.034)	−0.035 (0.024)
**Dinner**							
Middle	0.565 (0.601)	−0.065 (0.113)	0.033 (0.025)	0.054 (0.027)	−0.056 (0.616)	−0.19 (0.269)	−0.178 (0.109)
High	0.194 (0.778)	−0.204 (0.138)	0.064 * (0.03)	0.072 * (0.033)	0.212 (0.71)	−0.03 (0.305)	−0.066 (0.139)
Middle × time	0.027 (0.056)	0.017 (0.012)	0.002 (0.003)	0.002 (0.003)	0.046 (0.075)	0.039 (0.034)	**0.045** (0.024)
High × time	−0.015 (0.053)	0.019 (0.012)	−0.003 (0.003)	−0.001 (0.003)	−0.02 (0.071)	0.015 (0.032)	0.001 (0.023)

Linear random-effect models adjusted for time, center, age, sex, education level (and their interaction with time), energy intake, BMI, hypertension, cardiovascular history, stroke history, hypercholesterolemia, CES-D score, smoking status, Mediterranean-like diet score, and physical activity. Results showing a trend (*p* < 0.07) and significant results (* *p* < 0.05; ** *p* < 0.01) are in bold. *APOE4*, Apolipoprotein E ε4 allele; BVRT, Benton Visual Retention Test; FIRS, Free Immediate Recall Score; FDRS, Free Delayed Recall Score; IST, Isaacs Set Test; MMSE, Mini Mental State Examination; SE, standard error; TMT, Trail Making Test.

**Table 3:** Associations of glycemic load with cognitive change in *APOE4* carriers (12-year follow-up).

Glycemic Load	IST n = 401	BVRT n = 401	log(TMTA) n = 348	log(TMTB) n = 348	FIRS n = 278	FDRS n = 275	MMSE n = 409
	β (SE)	β (SE)	β (SE)	β (SE)	β (SE)	β (SE)	β (SE)
**Daily**							
Middle	−0.719 (1.402)	−0.049 (0.259)	0.04 (0.059)	−0.024 (0.065)	−0.018 (1.58)	−0.037 (0.706)	0.183 (0.266)
High	−1.476 (1.741)	0.115 (0.316)	0.034 (0.071)	−0.034 (0.077)	0.428 (1.811)	0.747 (0.799)	0.141 (0.332)
Middle × time	0.086 (0.134)	−0.009 (0.033)	0.002 (0.006)	−0.002 (0.007)	−0.033 (0.2)	0.03 (0.093)	−0.013 (0.062)
High × time	0.042 (0.131)	−0.031 (0.032)	0.007 (0.006)	0.001 (0.006)	−0.167 (0.196)	−0.103 (0.09)	−0.028 (0.062)
**Breakfast**							
Middle	−1.571 (1.237)	−0.055 (0.234)	0.037 (0.054)	−0.022 (0.058)	−0.771 (1.475)	−0.649 (0.654)	0.088 (0.238)
High	−1.647 (1.444)	−0.201 (0.267)	0.065 (0.059)	−0.06 (0.064)	0.192 (1.534)	−0.133 (0.677)	0.165 (0.279)
Middle × time	−0.11 (0.134)	−0.041 (0.034)	0.003 (0.007)	0.002 (0.007)	−0.095 (0.2)	−0.007 (0.092)	−0.143 * (0.061)
High × time	0.018 (0.126)	−0.053 (0.031)	0.002 (0.006)	0.004 (0.006)	−0.269 (0.18)	−0.076 (0.083)	−0.051 (0.057)
**Lunch**							
Middle	−0.772 (1.27)	0.006 (0.242)	**0.114** * (0.055)	−0.005 (0.06)	1.738 (1.521)	0.325 (0.683)	0.032 (0.246)
High	−0.202 (1.58)	0.219 (0.293)	0.085 (0.065)	−0.066 (0.07)	0.545 (1.674)	−0.452 (0.76)	−0.162 (0.302)
Middle × time	0.038 (0.132)	0.021 (0.033)	−0.01 (0.006)	−0.001 (0.006)	−0.09 (0.195)	−0.007 (0.089)	0.039 (0.06)
High × time	0.011 (0.131)	−0.024 (0.032)	−0.006 (0.006)	0.002 (0.006)	−0.082 (0.188)	−0.055 (0.087)	−0.024 (0.06)
**Afternoon snack**							
Middle	15.27 (8.8)	0.896 (1.499)	−0.032 (0.322)	0.082 (0.345)	−0.888 (6.569)	−1.579 (2.938)	−2.218 (1.747)
High	14.429 (8.908)	0.421 (1.524)	0.071 (0.326)	0.277 (0.349)	0.386 (6.723)	−1.213 (3.01)	−2.522 (1.77)
Middle × time	−0.069 (0.133)	−0.071 * (0.033)	0.004 (0.006)	0.005 (0.007)	0.081 (0.198)	−0.005 (0.091)	−0.04 (0.06)
High × time	−0.183 (0.129)	−0.04 (0.032)	0.011 (0.006)	0.002 (0.006)	**−0.343** (0.188)	−0.175 * (0.087)	−0.158 ** (0.059)
**Dinner**							
Middle	0.962 (1.343)	−0.012 (0.255)	−0.026 (0.058)	−0.051 (0.063)	1.418 (1.589)	0.351 (0.711)	−0.002 (0.255)
High	2.361 (1.765)	0.281 (0.31)	−0.011 (0.071)	−0.058 (0.077)	0.943 (1.81)	−0.116 (0.805)	−0.184 (0.32)
Middle × time	−0.067 (0.139)	−0.031 (0.035)	0.006 (0.007)	0.006 (0.007)	−0.141 (0.204)	−0.054 (0.094)	−0.034 (0.063)
High × time	0.029 (0.128)	−0.02 (0.032)	0.005 (0.006)	−0.004 (0.006)	−0.129 (0.193)	−0.033 (0.087)	0.012 (0.058)

Linear random-effect models adjusted for time, center, age, sex, education level (and their interaction with time), energy intake, BMI, hypertension, cardiovascular history, stroke history, hypercholesterolemia, CES-D score, smoking status, Mediterranean-like diet score, and physical activity. Results showing a trend (*p* < 0.07) and significant results (* *p* < 0.05; ** *p* < 0.01) are in bold. *APOE4*, Apolipoprotein E ε4 allele; BVRT, Benton Visual Retention Test; FIRS, Free Immediate Recall Score; FDRS, Free Delayed Recall Score; IST, Isaacs Set Test; MMSE, Mini Mental State Examination; SE, standard error; TMT, Trail Making Test.

## Supplementary Materials

The following are available online at https://www.mdpi.com/2072-6643/12/12/3619/s1, Table S1. Comparison of the characteristics of participants according to afternoon snack glycemic load, Table S2. Associations of glycemic load with cognitive changes during the 12-year follow-up, Table S3. Comparison of the characteristics of participants with and without incident dementia during the 12-year follow-up, Table S4. Associations of glycemic load with cognitive changes (12-year follow-up) in *APOE4* non-carriers without incident dementia, Table S5. Associations of glycemic load with cognitive changes (12-year follow-up) in *APOE4* carriers without incident dementia.
