# Supplementary material for: High Glycemic Load Is Associated with Cognitive Decline in Apolipoprotein E ε4 Allele Carriers
Source: Nutrients. 2020 Nov 25;12(12):3619. doi: 10.3390/nu12123619 (PMC7761247; doi:10.3390/nu12123619)
Supplement: Supplementary file 1 [file nutrients-12-03619-s001.pdf]

Supplementary Files

Table S1. Comparison of the characteristics of participants according to afternoon-snack glycemic load.

**Characteristics**<br>Mean (SD) or n (%)	**GL = 0**<br>n = 859	**GL > 0**<br>n = 1680	**P value**[1]
Montpellier center	301 (35)	958 (57)	<.0001
Age (years)	75.8 (4.9)	76 (4.9)	0.4496
Women	501 (58.3)	1098 (65.4)	0.0006
Education level			0.029
No school	245 (28.5)	410 (24.4)	
Primary school	252 (29.3)	474 (28.2)	
High school	193 (22.5)	392 (23.3)	
Graduated	167 (19.4)	400 (23.8)	
MMSE (IQR)	27.6 (1.8)	27.5 (1.8)	0.3808
APOE4 carrier	153 (17.8)	289 (17.2)	0.6259
BMI (kg/m^2)	25.8 (3.8)	25.3 (3.8)	0.0094
Hypertension	506 (58.9)	967 (57.6)	0.4797
Cardiovascular history	86 (10)	163 (9.7)	0.8592
Stroke history	33 (3.8)	68 (4)	0.8718
Hypercholesterolemia	472 (54.9)	919 (54.7)	0.4889
Depressive symptomatology[2]	77 (9)	123 (7.3)	0.1651
Tobacco use (current or past)	345 (40.2)	602 (35.8)	0.0365
Mediterranean-like diet	4.7 (1.6)	5 (1.6)	<.0001
Physical activity	257 (29.9)	585 (34.8)	0.0366
Energy intake (kJ/day)	4128.2 (1062.1)	5259.8 (1610)	<.0001

[1] Chi-square test for categorical variables, ANOVA or Kruskal-Wallis test for continuous variables.

[2] evaluated with the Center for Epidemiological Studies-Depression scale.

APOE4, Apolipoprotein E ε4 allele; BMI, Body Mass Index; IQR Interquartile range; MMSE, Mini Mental State Examination; SD, standard deviation.

Table S2. Associations of glycemic load with cognitive changes during the 12-year follow-up.

Glycemic load	IST n = 2337 β (SE)	BVRT n = 2314 β (SE)	log(TMTA) n = 2055 β (SE)	log(TMTB) n = 2032 β (SE)	FIRS n = 1703 β (SE)	FDRS n = 1697 β (SE)	MMSE n = 2362 β (SE)
**Daily**							
Middle	-0.467 (0.629)	-0.106 (0.118)	0.003 (0.026)	0.008 (0.028)	0.719 (0.648)	0.364 (0.284)	-0.11 (0.116)
High	-0.582 (0.766)	-0.116 (0.139)	0.023 (0.03)	-0.002 (0.033)	1.036 (0.73)	0.535 (0.317)	0.03 (0.14)
Middle x time	0.101 (0.056)	0.008 (0.012)	**-0.006*** (0.003)	-0.003 (0.003)	0.012 (0.077)	0.002 (0.035)	0.02 (0.025)
High x time	0.09 (0.055)	0.011 (0.012)	-0 .004 (0.003)	-0.002 (0.003)	0.044 (0.074)	0.004 (0.034)	-0.014 (0.024)
Middle x APOE4	1.177 (1.249)	0.124 (0.246)	-0.004 (0.003)	-0.025 (0.061)	-1.022 (1.432)	-0.696 (0.641)	0.34 (0.229)
High x APOE4	0.91 (1.225)	0.243 (0.241)	0.034 (0.053)	-0.023 (0.056)	-0.756 (1.401)	-0.161 (0.616)	0.197 (0.226)
Middle x time x APOE4	-0.021 (0.143)	-0.02 (0.031)	0.009 (0.051)	0.003 (0.007)	-0.066 (0.196)	0.029 (0.091)	-0.035 (0.062)
High x time x APOE4	-0.067 (0.139)	-0.044 (0.03)	0.008 (0.007)	0.003 (0.007)	-0.225 (0.192)	-0.098 (0.088)	-0.024 (0.063)
**Breakfast**							
Middle	0.465 (0.568)	0.041 (0.109)	-0.034 (0.023)	-0.033 (0.026)	-1.004 (0.604)	0.108 (0.269)	0.039 (0.104)
High	-0.065 (0.64)	-0.039 (0.119)	0.012 (0.026)	-0.039 (0.028)	-0.35 (0.633)	0.025 (0.279)	0.041 (0.117)
Middle x time	0.03 (0.057)	-0.005 (0.013)	0 (0.003)	0.002 (0.003)	0.136 (0.078)	-0.014 (0.036)	0.022 (0.025)
High x time	0.079 (0.054)	0.015 (0.012)	-0.003 (0.003)	0 (0.003)	0.087 (0.072)	0.008 (0.033)	0.022 (0.023)
Middle x APOE4	-1.331 (1.268)	0.057 (0.247)	0.08 (0.054)	0.04 (0.06)	0.38 (1.428)	-0.57 (0.64)	0.112 (0.234)
High x APOE4	-0.755 (1.193)	0.14 (0.232)	0.051 (0.049)	0.003 (0.055)	0.56 (1.326)	-0.13 (0.587)	0.234 (0.222)
Middle x time x APOE4	-0.132 (0.142)	-0.049 (0.031)	0.002 (0.007)	0 (0.007)	-0.23 (0.196)	0.02 (0.09)	**-0.161**** (0.061)
High x time x APOE4	-0.061 (0.135)	**-0.072*** (0.029)	0.005 (0.006)	0.003 (0.006)	**-0.338** (0.177)	-0.066 (0.082)	-0.073 (0.057)
**Lunch**							
Middle	**-1.328*** (0.584)	-0.055 (0.112)	0.031 (0.024)	**0.069**** (0.027)	0.265 (0.623)	-0.272 (0.275)	0.022 (0.107)
High	**-1.923**** (0.693)	**-0.275*** (0.127)	**0.074** ** (0.028)	**0.063*** (0.03)	0.206 (0.67)	-0.051 (0.293)	-0.036 (0.127)
Middle x time	0.017 (0.057)	-0.007 (0.013)	-0.001 (0.003)	**-0.008**** (0.003)	-0.061 (0.078)	0.024 (0.036)	-0.022 (0.025)
High x time	**0.103** (0.055)	0.011 (0.012)	**-0.006*** (0.003)	**-0.006*** (0.003)	0.058 (0.073)	0.022 (0.033)	-0.012 (0.024)

Table S2. Associations of glycemic load with cognitive changes during the 12-year follow-up *(Continued).*

Middle x APOE4	0.672 (1.234)	-0.004 (0.249)	0.058 (0.054)	-0.065 (0.059)	1.515 (1.435)	0.722 (0.63)	-0.004 (0.234)
High x APOE4	1.627 (1.216)	0.293 (0.238)	0.008 (0.051)	-0.094 (0.057)	-0.312 (1.329)	-0.532 (0.597)	-0.162 (0.226)
Middle x time x APOE4	0.039 (0.142)	0.025 (0.031)	-0.01 (0.007)	0.007 (0.007)	-0.016 (0.192)	-0.033 (0.088)	0.057 (0.06)
High x time x APOE4	-0.076 (0.138)	-0.038 (0.03)	-0.001 (0.006)	0.009 (0.007)	-0.115 (0.183)	-0.054 (0.085)	-0.009 (0.06)
**Afternoon snack**							
Middle	2.151 (4.872)	-0.187 (0.813)	0.044 (0.176)	0.035 (0.185)	-1.347 (3.4)	-1.347 (3.4)	-0.579 (0.875)
High	1.184 (4.913)	-0.256 (0.82)	0.075 (0.178)	0.067 (0.187)	-1.619 (3.437)	-1.619 (3.437)	-0.743 (0.883)
Middle x time	-0.051 (0.055)	-0.006 (0.012)	-0.002 (0.003)	-0.002 (0.003)	0.022 (0.074)	0.022 (0.074)	-0.016 (0.024)
High x time	0.046 (0.056)	-0.006 (0.012)	-0.002 (0.003)	0 (0.003)	0.104 (0.075)	0.104 (0.075)	-0.033 (0.024)
Middle x APOE4	0.381 (1.197)	0.181 (0.242)	-0.014 (0.051)	-0.071 (0.057)	-1.914 (1.39)	-1.914 (1.39)	-0.261 (0.223)
High x APOE4	1.091 (1.163)	-0.219 (0.235)	0.018 (0.05)	0.058 (0.055)	0.248 (1.322)	0.248 (1.322)	-0.153 (0.224)
Middle x time x APOE4	-0.051 (0.138)	**-0.056** (0.03)	0.006 (0.006)	0.007 (0.007)	0.081 (0.191)	0.081 (0.191)	-0.021 (0.059)
High x time x APOE4	**-0.281*** (0.132)	-0.021 (0.029)	**0.014*** (0.006)	0.002 (0.007)	**-0.437*** (0.178)	**-0.148** (0.082)	**-0.13*** (0.057)
**Dinner**							
Middle	0.572 (0.586)	-0.069 (0.111)	0.03 (0.024)	**0.049** (0.026)	-0.03 (0.613)	-0.03 (0.613)	**-0.217*** (0.107)
High	0.196 (0.726)	-0.191 (0.132)	**0.059*** (0.029)	**0.066*** (0.032)	0.269 (0.691)	0.269 (0.691)	-0.155 (0.133)
Middle x time	0.024 (0.057)	0.016 (0.012)	0.002 (0.003)	0.002 (0.003)	0.045 (0.076)	0.045 (0.076)	0.043 (0.025)
High x time	-0.017 (0.054)	0.018 (0.012)	-0.003 (0.003)	-0.001 (0.003)	-0.021 (0.072)	-0.021 (0.072)	0 (0.023)
Middle x APOE4	0.279 (1.289)	0.043 (0.251)	-0.035 (0.054)	-0.052 (0.059)	1.146 (1.444)	1.146 (1.444)	0.355 (0.237)
High x APOE4	**2.261** (1.185)	0.337 (0.231)	-0.029 (0.049)	-0.062 (0.056)	0.795 (1.35)	0.795 (1.35)	0.291 (0.216)
Middle x time x APOE4	-0.079 (0.147)	-0.047 (0.032)	0.005 (0.007)	0.003 (0.007)	-0.199 (0.198)	-0.199 (0.198)	-0.071 (0.063)
High x time x APOE4	0.068 (0.137)	-0.037 (0.029)	0.007 (0.006)	-0.005 (0.007)	-0.112 (0.186)	-0.112 (0.186)	0.021 (0.059)

Linear random-effect models adjusted for time, center, age, sex, education level, and *APOE4* (and their interaction with time), energy intake, BMI, hypertension, cardiovascular history, stroke history, hypercholesterolemia, CES-D, smoking status, Mediterranean-like diet score, and physical activity.

Results showing a trend (**P<0.07**) and significant results (*** P <0.05**; **** P <0.01**; ***** P <0.001**) are in bold.

APOE4, Apolipoprotein E ε4 allele; BVRT, Benton Visual Retention Test; FIRS, Free Immediate Recall Score; FDRS, Free Delayed Recall Score; IST, Isaacs Set Test; MMSE, Mini Mental State Examination; SE, standard error; TMT, Trail Making Test

Table S3. Comparison of the characteristics of participants with and without incident dementia during the 12-year follow-up.

**Characteristics**<br>Mean (SD) or n (%)	**Dementia-free**<br>n = 2202	**Incident dementia**<br>n = 337	**P value**[1]
Montpellier center	1150 (52.2)	109 (32.3)	<.0001
Age (years)	75.6 (4.8)	78.1 (4.6)	<.0001
Women	1360 (61.8)	239 (70.9)	0.0015
Education level			0.0324
No school	547 (24.8)	108 (32)	
Primary school	632 (28.7)	94 (27.9)	
High school	519 (23.6)	66 (19.6)	
Graduated	499 (22.7)	68 (20.2)	
MMSE (IQR)	28 (27-29)	27 (26-28)	<.0001
APOE4 carrier	361 (16.4)	81 (24)	<.001
BMI (kg/m^2)	25.5 (3.8)	25.4 (3.8)	0.8445
Type 2 diabetes	1273 (57.8)	200 (59.3)	0.6065
Hypertension	216 (9.8)	33 (9.8)	1
Cardiovascular history	84 (3.8)	17 (5)	0.3557
Stroke history	1193 (54.2)	198 (58.8)	0.0616
Hypercholesterolemia	142 (6.4)	21 (6.2)	0.9743
Depressive symptomatology[2]	163 (7.4)	37 (11)	0.03
Tobacco use (current or past)	843 (38.3)	104 (30.9)	0.0104
Mediterranean-like diet	5 (1.6)	4.7 (1.5)	0.0236
Physical activity	745 (33.8)	97 (28.8)	0.4353
Glycemic load (/day)	109.3 (34.4)	109.3 (35.1)	0.9983
Energy intake (kJ/day)	4882.1 (1541.5)	4843.4 (1559.4)	0.6683

[1] Chi-square test for categorical variables, ANOVA or Kruskal-Wallis test for continuous variables.

[2] evaluated with the Center for Epidemiological Studies-Depression scale.

APOE4, Apolipoprotein E ε4 allele; BMI, Body Mass Index; IQR Interquartile range; MMSE, Mini Mental State Examination; SD, standard deviation.

Table S4. Associations of glycemic load with cognitive changes (12-year follow-up) in *APOE4* non-carriers without incident dementia.

Glycemic load	IST n = 1598 β (SE)	BVRT n = 1583 β (SE)	log(TMTA) n = 1431 β (SE)	log(TMTB) n = 1428 β (SE)	FIRS n = 1228 β (SE)	FDRS n = 1227 β (SE)	MMSE n = 1605 β (SE)
**Daily**							
Middle	-0.368 (0.684)	-0.066 (0.117)	0.01 (0.027)	0.017 (0.029)	0.333 (0.654)	0.08 (0.28)	-0.113 (0.119)
High	-0.357 (0.852)	-0.124 (0.141)	0.027 (0.033)	0.016 (0.036)	0.605 (0.75)	0.112 (0.317)	-0.124 (0.148)
Middle x time	0.088 (0.05)	0.007 (0.012)	**-0.005***(0.003)	-0.004 (0.003)	0.033 (0.072)	0.011 (0.032)	**0.023**(0.012)
High x time	0.097 (0.05)	0.015 (0.012)	**-0.006***(0.002)	-0.003 (0.003)	0.093 (0.07)	0.03 (0.031)	0.002 (0.012)
**Breakfast**							
Middle	0.465 (0.617)	0.05 (0.107)	-0.019 (0.024)	-0.024 (0.027)	-0.804 (0.607)	0.17 (0.266)	0.093 (0.105)
High	0.003 (0.702)	0.023 (0.119)	0.011 (0.027)	-0.039 (0.03)	-0.374 (0.638)	-0.072 (0.276)	0.129 (0.119)
Middle x time	-0.018 (0.052)	-0.006 (0.013)	-0.002 (0.003)	0 (0.003)	0.118 (0.072)	-0.024 (0.033)	0.006 (0.013)
High x time	0.053 (0.049)	0.018 (0.012)	-0.003 (0.002)	0.001 (0.003)	0.076 (0.067)	-0.001 (0.03)	0.011 (0.012)
**Lunch**							
Middle	-0.93 (0.635)	-0.007 (0.112)	0.014 (0.026)	**0.056***(0.028)	0.15 (0.629)	-0.438 (0.271)	-0.031 (0.11)
High	**-1.46**(0.759)	-0.232 (0.128)	**0.062***(0.029)	**0.061**(0.032)	-0.114 (0.682)	-0.321 (0.292)	-0.136 (0.131)
Middle x time	0.022 (0.051)	-0.012 (0.013)	0.002 (0.003)	**-0.005**(0.003)	-0.046 (0.073)	0.039 (0.033)	0.007 (0.013)
High x time	0.069 (0.048)	0.004 (0.012)	**-0.005**(0.002)	**-0.005***(0.003)	0.07 (0.068)	0.034 (0.03)	0.005 (0.012)
**Afternoon snack**							
Middle	-1.675 (5.617)	-0.257 (0.855)	-0.051 (0.2)	-0.101 (0.216)	-3.126 (3.789)	0.188 (1.559)	-0.283 (0.955)
High	-2.538 (5.664)	-0.362 (0.863)	-0.004 (0.202)	-0.073 (0.218)	-3.362 (3.826)	0.37 (1.575)	-0.468 (0.963)
Middle x time	-0.008 (0.049)	-0.005 (0.012)	-0.004 (0.002)	-0.003 (0.003)	0.031 (0.07)	0.005 (0.031)	-0.008 (0.012)
High x time	0.081 (0.05)	-0.001 (0.012)	-0.004 (0.003)	-0.001 (0.003)	0.097 (0.071)	0.018 (0.032)	-0.006 (0.012)
**Dinner**							
Middle	-0.108 (0.636)	-0.168 (0.11)	0.044 (0.025)	**0.071****(0.027)	-0.406 (0.617)	-0.35 (0.266)	-0.191 (0.109)
High	-0.5 (0.809)	**-0.288***(0.133)	**0.066***(0.031)	**0.09****(0.033)	-0.021 (0.699)	-0.133 (0.298)	0.036 (0.137)
Middle x time	0.001 (0.051)	0.016 (0.012)	0.001 (0.003)	0.003 (0.003)	0.074 (0.071)	0.054 (0.031)	0.019 (0.012)
High x time	0.016 (0.048)	**0.023***(0.012)	-0.004 (0.002)	-0.003 (0.003)	0.017 (0.068)	0.03 (0.03)	0 (0.012)

Linear random-effect models adjusted for time, center, age, sex, and education level (and their interaction with time), energy intake, BMI, hypertension, cardiovascular history, stroke history, hypercholesterolemia, CES-D, smoking status, Mediterranean-like diet score, and physical activity.
Results showing a trend (**P<0.07**) and significant results (* **P <0.05**; ** **P <0.01**; *** **P <0.001**) are in bold.
APOE4, Apolipoprotein E ε4 allele; BVRT, Benton Visual Retention Test; FIRS, Free Immediate Recall Score; FDRS, Free Delayed Recall Score; IST, Isaacs Set Test; MMSE, Mini Mental State Examination; SE, standard error; TMT, Trail Making Test

Table S5. Associations of glycemic load with cognitive changes (12-year follow-up) in *APOE4* carriers without incident dementia.

Glycemic load	**IST** n = 323 β (SE)	**BVRT** n = 324 β (SE)	**log(TMTA)** n = 283 β (SE)	**log(TMTB)** n = 287 β (SE)	**FIRS** n = 242 β (SE)	**FDRS** n = 249 β (SE)	**MMSE** n = 409 β (SE)
**Daily**							
Middle	-0.916 (1.453)	-0.134 (0.24)	0.036 (0.061)	-0.026 (0.068)	0.546 (1.583)	0.094 (0.687)	0.262 (0.264)
High	-1.166 (1.811)	0.026 (0.29)	0.053 (0.074)	-0.052 (0.081)	0.195 (1.767)	0.533 (0.758)	0.197 (0.324)
Middle x time	0.085 (0.125)	0.019 (0.032)	-0.001 (0.007)	-0.001 (0.007)	-0.07 (0.193)	0.025 (0.085)	0.015 (0.029)
High x time	0.052 (0.123)	-0.013 (0.032)	0.003 (0.006)	0.004 (0.006)	-0.125 (0.19)	-0.071 (0.083)	0.02 (0.029)
**Breakfast**							
Middle	-2.121 (1.328)	-0.189 (0.228)	0.041 (0.058)	-0.011 (0.063)	-0.935 (1.518)	-0.709 (0.65)	-0.058 (0.254)
High	-2.176 (1.5)	-0.159 (0.248)	0.032 (0.062)	-0.067 (0.068)	1.112 (1.523)	0.546 (0.651)	0.133 (0.284)
Middle x time	0.009 (0.126)	-0.011 (0.033)	-0.003 (0.007)	0 (0.007)	-0.005 (0.196)	0.056 (0.087)	-0.037 (0.03)
High x time	0.068 (0.116)	-0.035 (0.03)	0 (0.006)	0.002 (0.006)	-0.286 (0.173)	-0.094 (0.076)	-0.015 (0.028)
**Lunch**							
Middle	-0.513 (1.412)	-0.098 (0.237)	0.101 (0.062)	0.051 (0.066)	1.752 (1.52)	0.163 (0.662)	0.06 (0.262)
High	0.031 (1.697)	-0.006 (0.277)	0.073 (0.07)	-0.038 (0.077)	-0.115 (1.65)	-0.53 (0.726)	-0.31 (0.307)
Middle x time	0.012 (0.125)	0.008 (0.033)	**-0.012** (0.007)	-0.002 (0.007)	-0.239 (0.19)	-0.046 (0.083)	-0.016 (0.029)
High x time	0.041 (0.123)	0.002 (0.032)	**-0.013***(0.006)	0.004 (0.007)	-0.036 (0.185)	-0.039 (0.081)	-0.003 (0.029)

Table S5. Associations of glycemic load with cognitive changes (12-year follow-up) in *APOE4* carriers without incident dementia *(Continued)*.

**Afternoon snack**							
Middle	14.505 (8.293)	1.198 (1.236)	-0.04 (0.309)	0.028 (0.34)	-1.805 (5.62)	-2.272 (2.449)	-2.226 (1.487)
High	13.994 (8.414)	0.769 (1.257)	0.051 (0.313)	0.167 (0.344)	-0.477 (5.736)	-1.862 (2.504)	-2.503 (1.511)
Middle x time	0.081 (0.122)	-0.039 (0.032)	-0.006 (0.006)	0.004 (0.007)	0.131 (0.194)	0.048 (0.084)	0.045 (0.029)
High x time	-0.082 (0.12)	-0.026 (0.031)	0.002 (0.007)	0.002 (0.006)	-0.221 (0.187)	-0.072 (0.082)	0.013 (0.029)
**Dinner**							
Middle	1.396 (1.451)	0.075 (0.252)	0.001 (0.061)	-0.059 (0.068)	0.594 (1.595)	0.294 (0.693)	0.108 (0.276)
High	2.649 (1.82)	0.066 (0.292)	-0.04 (0.073)	-0.135 (0.081)	0.545 (1.719)	-0.247 (0.745)	0.026 (0.325)
Middle x time	0.003 (0.131)	-0.035 (0.034)	0.009 (0.007)	0.006 (0.007)	0.01 (0.199)	-0.005 (0.087)	-0.027 (0.031)
High x time	0.058 (0.119)	-0.008 (0.032)	0.006 (0.006)	0 (0.007)	-0.028 (0.187)	0.023 (0.081)	-0.025 (0.029)

Linear random-effect models adjusted for time, center, age, sex, and education level (and their interaction with time), energy intake, BMI, hypertension, cardiovascular history, stroke history, hypercholesterolemia, CES-D, smoking status, Mediterranean-like diet score, and physical activity.

Results showing a trend (**P<0.07**) and significant results (*** P <0.05**; **** P <0.01**; ***** P <0.001**) are in bold.

APOE4, Apolipoprotein E ε4 allele; BVRT, Benton Visual Retention Test; FIRS, Free Immediate Recall Score; FDRS, Free Delayed Recall Score; IST, Isaacs Set Test; MMSE, Mini Mental State Examination; SE, standard error; TMT, Trail Making Test
